# Practical considerations for managing patients being treated with topical roflumilast

**DOI:** 10.3389/jpps.2026.16026

**Published:** 2026-05-08

**Authors:** Sameh Hanna, Ahmad Chehade, Christina Han, Susan Poelman, Ravina Sanghera, Carolyn Whiskin, Aaron Sihota

**Affiliations:** 1 Dermatology on Bloor, Toronto, ON, Canada; 2 Division of Dermatology, Temerty School of Medicine, University of Toronto, Toronto, ON, Canada; 3 UPharmacy, Edmonton, AB, Canada; 4 Department of Dermatology and Skin Science, University of British Columbia, Vancouver, BC, Canada; 5 Division of Dermatology, University of Calgary, Calgary, AB, Canada; 6 Beacon Dermatology, Calgary, AB, Canada; 7 Faculty of Pharmacy and Pharmaceutical Sciences, University of Alberta, Edmonton, AB, Canada; 8 Charlton Health Inc., Hamilton, ON, Canada; 9 Brant Arts Pharmacy, Burlington, ON, Canada; 10 University of Toronto, Toronto, ON, Canada; 11 Polaris Health, Vancouver, BC, Canada; 12 Faculty of Pharmaceutical Sciences, University of British Columbia, Vancouver, BC, Canada

**Keywords:** adverse events, inflammatory dermatoses, PDE4 inhibitor, pharmacokinetics, roflumilast

## Abstract

Topical roflumilast, available in 0.3% cream, 0.15% cream, and 0.3% foam formulations, is a novel, highly potent phosphodiesterase-4 (PDE4) inhibitor that was recently approved in Canada for the treatment of plaque psoriasis, atopic dermatitis, and seborrheic dermatitis, respectively. Topical formulations were shown in clinical trials to significantly improve disease symptoms with excellent tolerability and minimal adverse events. An oral form of roflumilast has also been approved in Canada since 2010 for the maintenance treatment of chronic obstructive pulmonary disease. The product monograph for topical roflumilast currently references a substantial amount of safety information specific to the oral formulation due to a lack of long-term safety data. Furthermore, no formal drug-drug interaction studies have been conducted with topical roflumilast, nor in special populations in whom oral roflumilast is contraindicated. Due to the impact that the route of administration has on the pharmacokinetic profile of a drug and its subsequent ability to produce undesirable or toxic effects, this presents a challenge for pharmacists who are tasked with determining how to best interpret and communicate this safety information to patients. This paper reviews the safety information contained within the product monograph for topical roflumilast in the context of the pharmacokinetic differences between the topical and oral formulations. The impact of the route of administration on adverse effect risk, drug-drug interactions, and contraindications is highlighted, and practical guidance is provided to assist pharmacists in interpreting and applying this information in practice.

## Introduction

Topical medications are often derived from oral formulations. Topicals have the benefit of delivering active agents directly to the affected area while reducing absorption into systemic circulation, avoiding hepatic metabolism, and subsequently minimizing potential systemic adverse effects (AEs) [1, 2]. However, the safety profile of a topical formulation is based, at least in part, on initial studies of the oral formulation. Pharmacists play a key role in ensuring that patients are appropriately informed of AEs, contraindications, and interactions associated with each formulation of a given drug, and the route of administration is an important consideration when counselling patients on the potential safety risks [3, 4]. When faced with medications that have both oral and topical formulations, discussing the safety profile within the context of the route of administration is necessary.

The purpose of this paper is to review safety information in the context of the pharmacokinetic and pharmacodynamic differences between the oral and topical formulations of roflumilast. The impacts of the route of administration on AE risk, drug-drug interactions, and contraindications are highlighted, and practical guidance is provided to assist pharmacists in interpreting and applying this information in practice.

This narrative review was informed by targeted literature searches of PubMed and relevant product monographs to identify clinical trials, pharmacokinetic studies, and safety data related to oral and topical roflumilast. Priority was given to phase 2/3 trials, regulatory documents, and key pharmacology studies. Given the emerging nature of the topical formulation, the review is not systematic but aims to synthesize currently available evidence relevant to clinical practice.

### Oral vs. topical formulations

Findings from a post-marketing safety study of oral tofacitinib [5], a Janus kinase (JAK) inhibitor approved for rheumatoid arthritis (RA), led to boxed warnings for multiple severe systemic AEs being added to all oral JAK inhibitors indicated for the treatment of RA and/or other inflammatory conditions, as well as ruxolitinib, a topical JAK inhibitor approved for the treatment of nonsegmental vitiligo and mild-to-moderate atopic dermatitis [6]. However, a recent analysis of real-world safety data showed that the AEs contained within these boxed warnings, including the risk of serious infections, malignancies, thrombosis, and major adverse cardiovascular events, were not reported with the topical ruxolitinib use during the first year following approval (Supplementary Table S1) [7]. Topical tacrolimus, a calcineurin inhibitor used for the treatment of atopic dermatitis, previously contained a black box warning for increased risk of malignancy based on the systemic immunosuppression associated with the use of oral calcineurin inhibitors. This warning was subsequently removed following the publication of findings from two large, long-term, non-interventional, post-authorization safety studies that demonstrated a low risk of malignancy with topical use [8, 9]. The oral form of diclofenac, a non-steroidal anti-inflammatory drug, is associated with an increased risk of gastrointestinal and cardiovascular AEs, as well as renal and hepatic toxicity, all of which were shown to be lower with topical use, likely owing to decreased systemic exposure [10]. Likewise, the oral form of dapsone, an antibacterial agent used to treat infectious conditions such as leprosy and mycetoma, is associated with an increased risk of hemolytic anemia; however, no such risk was linked to the topical formulation used to treat acne vulgaris [11]. These examples illustrate that key differences can exist in the safety profiles of drugs with different routes of administration. They also highlight the importance of ongoing pharmacovigilance to optimize the safety profiles of drugs as new formulations are developed.

Roflumilast is another, more recent example of a drug with both topical and oral formulations. Indicated for inflammatory skin and respiratory conditions, it is a non-steroidal anti-inflammatory agent that selectively inhibits phosphodiesterase-4 (PDE4), an enzyme involved in the degradation of cyclic adenosine monophosphate (cAMP). The increase in cAMP levels subsequently suppresses pro-inflammatory mediators and reduces inflammatory cytokines [12]. The oral formulation, available as a 500-mcg tablet, was approved by Health Canada on November 23, 2010, for the maintenance treatment of severe chronic obstructive pulmonary disease (COPD) based on efficacy and safety results from six phase 3 clinical trials [13–15]. More recently, topical roflumilast was approved for multiple dermatologic conditions in Canada [16]. It is indicated for the treatment of plaque psoriasis in patients ≥12 years of age (0.3% cream; 0.3% foam), mild to moderate atopic dermatitis in patients ≥6 years of age (0.15% cream), and seborrheic dermatitis in patients ≥9 years of age (0.3% foam) [16]. These approvals were based on data from the DERMIS-1/DERMIS-2 phase 3 trials for psoriasis [17], INTEGUMENT-1/INTEGUMENT-2 phase 3 trials for atopic dermatitis [18], and a phase 2a clinical trial [19] and STRATUM phase 3 clinical trial [20] for seborrheic dermatitis.

Topical roflumilast cream (0.3% and 0.15%) and foam 0.3%, were shown in randomized clinical trials to be safe and well-tolerated with minimal AEs. The most common AEs reported were diarrhea, headache, nausea, and nasopharyngitis, though these were relatively infrequent, occurring in no more than 3.1% of study participants, and were predominantly mild to moderate in severity [17, 20] (Supplementary Table S2). However, given that the topical formulations were only recently approved, there is currently a paucity of long-term safety data with real-world use. Further, no formal drug-drug interaction studies have been conducted with topical roflumilast, nor in special populations in whom oral roflumilast is contraindicated. Due to the lack of safety data specific to topical use, a large portion of the safety information for oral roflumilast has been carried over to the product monograph for topical roflumilast. Considering the impact that the route of administration has on the bioavailability of drug, which is considerably higher with oral roflumilast (79%) [21] compared to topical roflumilast cream (0.3%, 1.5%) [22], this presents a challenge for pharmacists, who are tasked with determining how to best interpret and communicate this safety information to patients.

Given the substantial amount of safety information derived from the oral roflumilast clinical trials contained within the product monograph for topical roflumilast, this paper aims to support practicing pharmacists in their discussions with patients being treated with topical roflumilast.

### Comparison of adverse events between oral and topical roflumilast

Despite some overlap in the AE profiles of oral and topical roflumilast, there are notable differences (Supplementary Table S2). The most common adverse drug reactions associated with roflumilast cream 0.3% were diarrhea (3.1%), headache (2.4%), insomnia (1.4%), nausea (1.2%), upper respiratory (1.0%) and urinary tract (1.0%) infections, and application site pain (1.0%) [16, 17], while the most common AEs reported with application of the 0.15% cream were headache (2.9%), nausea (1.9%), diarrhea (1.5%), vomiting (1.5%), and application site pain (1.5%) [16, 18]. Weight loss (≥5% body weight) was reported in 4% of patients treated with roflumilast cream 0.3% but was determined to be not clinically meaningful [16, 23]. The most common AEs associated with roflumilast foam 0.3% were nasopharyngitis (1.5%), nausea (1.3%), and headache (1.1%) [16, 20]. The majority of AEs were mild to moderate in severity and led to treatment discontinuation in only 1.0% of patients treated with roflumilast cream (0.3%) [17], 1.6% of patients treated with 0.15% cream [18], and 0.9% of patients treated with roflumilast foam [19, 20]. Additionally, most of the reported cases of AEs, such as diarrhea and nausea, were only observed within the first 2 weeks of treatment and resolved over time without treatment interruption or the need for dose adjustment [16, 17]. Vehicle-treated patients have similar or slightly lower rates of adverse drug reactions compared with topical roflumilast-treated patients [16].

Many of the AEs associated with topical application, particularly those associated with the gastrointestinal tract, were also reported in COPD patients treated with oral roflumilast, though rates were higher with oral use than those observed with both topical formulations. Other gastrointestinal AEs, including abdominal pain and gastritis, were only reported with oral administration [13–15, 24, 25]. In addition to these differences, oral roflumilast is associated with several systemic AEs not observed with the use of either topical formulation, including cardiac events (e.g., supra-ventricular arrhythmia), neurologic events (e.g., dizziness and tremors), and psychiatric events (e.g., anxiety and depression) [13–15, 24, 25]. Despite having only been reported with oral administration, these AEs have been included in the product monograph for topical roflumilast in addition to those specific to topical use [16] (Supplementary Table S2). This information may be alarming to patients prescribed topical roflumilast and could subsequently lead to concerns about its safety and tolerability; however, discussing the risk of AEs in the context of the route of administration can help pharmacists alleviate these concerns.

### Tolerability

A concern surrounding topical agents vs. the oral counterpart is risk for skin irritation. In the DERMIS-1 and -2 clinical trials, 98.8% and 98.6% of roflumilast-treated patients and 97.7% and 98.4% of vehicle-treated patients had no signs of irritation by weeks 4 and 8, respectively, as reported by the investigator. In addition, 99.3% and 98.8% of patients reported no or mild sensation after applying roflumilast cream by week 4 and week 8, respectively, which was consistent with vehicle cream (98.8% and 98.7% by week 4 and week 8, respectively). The vehicle for topical roflumilast therapies was designed at a physiological skin pH of 5.5. It is free from common irritants such as propylene glycol, polyethylene glycol, ethanol, and fragrances. The vehicle’s composition includes 50% water and an ultra-mild emulsifier, which results in no functional surfactant activity during clinical use. These properties may contribute to the tolerability profile of topical roflumilast reported in the clinical trials.

### Drug-drug interactions

The product monograph for topical roflumilast lists several established or potential drug-drug interactions; however, these are based on studies with oral roflumilast use, as no formal drug-drug interaction studies have been conducted with topical use [16]. These interaction data are derived from oral roflumilast studies and have not been directly evaluated with topical formulations. These studies show that, due to the metabolism of oral roflumilast by cytochrome P450 isozymes CYP3A4 and CYP1A2, co-administration with systemic CYP3A4 inhibitors (e.g., erythromycin, ketoconazole), CYP1A2 inhibitors (e.g., fluvoxamine), or dual inhibitors (e.g., enoxacin, cimetidine) may increase systemic exposure to roflumilast and consequently lead to an increase in the risk of AEs [26–30]. While these interactions should not be ignored, it is important to consider them in the context of the route of administration and the fact that available evidence is based on oral exposure. A topically applied medication may not achieve adequate systemic concentrations to interact with drugs taken orally, thus limiting or even circumventing any potential risk of co-administration [31]. Due to the low systemic absorption of topical roflumilast, which is discussed in more detail below, the expectation is that these interactions will be limited, although this has not been formally studied. However, until more data is available, patients should be advised that co-administering topical roflumilast with CYP3A4 inhibitors, CYP1A2 inhibitors, or dual CYP3A4/CYP1A2 inhibitors has the potential to increase their risk of experiencing an AE.

### Hepatic impairment as a contraindication

Although no studies have been performed with topical roflumilast in patients with hepatic insufficiency, the current product monograph states that its use is contraindicated in patients with moderate-to-severe hepatic impairment (Child-Pugh B or C) [16]. This recommendation is based on a pharmacokinetic study of oral roflumilast which found that a 250 mcg once daily dose increased total PDE4 inhibition by approximately 20% and 90% in patients with mild (Child-Pugh A) to moderate (Child-Pugh B) hepatic insufficiency, respectively, compared to healthy control subjects [32] and has not been directly evaluated with topical formulations.

Despite the absence of data specific to the topical formulation and given that current recommendations are extrapolated from oral data, the authors agree that its use is not advised in patients with moderate-to-severe hepatic impairment and recommend proceeding with caution in patients with only mild impairment. Practical guidance for addressing concerns about the use of topical roflumilast in the context of hepatic impairment is provided in Table 1.

**TABLE 1:** Practical guidance for addressing potential patient and pharmacist concerns about the safety and tolerability of topical roflumilast.

Patient/Pharmacist FAQ	Practical guidance
Common adverse events	
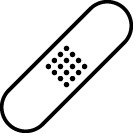 What are the common AEs of topical roflumilast? Are the cream and foam formulations associated with the same AEs?	• Roflumilast cream 0.3%, cream 0.15%, and foam 0.3% have similar AE profiles, though notable differences exist • 0.3% cream: AEs reported in the product monograph (any grade) include diarrhea (3.1%), headache (2.4%), insomnia (1.4%), nausea (1.2%), upper respiratory (1.0%) and urinary tract (1.0%) infections, and application site pain (1.0%) [16] • 0.15% cream: AEs reported in the product monograph (any grade) include headache (2.9%), nausea (1.9%), diarrhea (1.5%), vomiting (1.5%), and application site pain (1.5%) [16] • 0.3% foam: AEs reported in the product monograph (any grade) include nasopharyngitis (1.5%), nausea (1.3%), and headache (1.1%) [16] • Headache has been reported in clinical trials of topical roflumilast [16]. While the incidence is low and events are generally mild to moderate in severity, a causal relationship to treatment cannot be excluded. Other contributing factors may be considered in clinical assessment. Patients should be counselled that headache is a potential adverse event, and clinical judgement should guide management if symptoms occur • Application site symptoms are infrequent (reported in ≤1% of patients treated with either 0.3% cream or 0.3% foam and 1.5% of patients treated with 0.15% cream) [16]. If skin irritation occurs, the authors suggest refrigerating topical roflumilast for 15 min prior to application [expert opinion]
Serious adverse events	
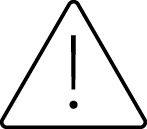 Are there any serious AEs I should know when using topical roflumilast?	• All serious AEs reported in clinical trials were considered unrelated to topical roflumilast use [17, 19, 20]
Gastrointestinal adverse events	
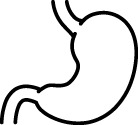 How should I counsel my patient about the risk of diarrhea with topical roflumilast?	• Diarrhea is an infrequent event with topical roflumilast use, occurring in only ∼3% of patients treated with 0.3% cream [17], 1.5% of patients treated with 0.15% cream [18], and <1% of patients treated with 0.3% foam [19, 20] • Diarrhea may be more problematic if it occurs in the geriatric population than in younger patients [expert opinion] • Based on real-world use, the authors note that patients are more likely to experience loose stools rather than true diarrhea [expert opinion] • Patients can be reassured that if diarrhea or loose stools occur, it is typically at the start of treatment, is most often mild, and generally resolves on its own without the need to interrupt treatment or adjust dosage [16]
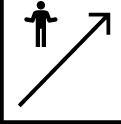 If my patient has a BSA of 1% vs. 3%, what consideration(s) should my counselling include (i.e., risk of loose stools vs. diarrhea)?	• Diarrhea has been reported more frequently in patients with higher baseline psoriasis-affected body surface area (BSA) treated with roflumilast cream 0.3% [pooled diarrhea rates: <5% BSA = 3 (1.1%); 5–10% BSA = 8 (3.9%); BSA >10% = 7 (6.7%)], likely due to application over a broader area [17, 33] • It is important to consider the patient’s affected BSA and corresponding extent of application area when counselling patients on the risk of diarrhea and other common AEs [expert opinion] • While there is no reported restriction on the maximum body area for application, prescribers should consider BSA when determining the dosage to optimize efficacy while minimizing risks [expert opinion]
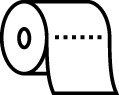 How should I manage diarrhea if it occurs?	• Diarrhea is typically mild and resolves on its own [16] • If it becomes moderate or persists, it can be managed by increasing fiber intake or, if necessary, using oral antidiarrheals such as loperamide [expert opinion]
Other safety considerations	
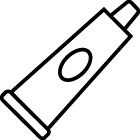 Is there a risk of systemic side effects with topical roflumilast, especially given its application on the skin?	• Systemic AEs, such as diarrhea and nausea, can occur with topical roflumilast, but its low systemic absorption decreases their likelihood of occurring and minimizes their severity if they do occur. Beyond the systemic AEs noted above, none of those observed with oral roflumilast (e.g., supraventricular tachycardia, dizziness, anxiety, depression) have been observed in clinical trials of topical roflumilast [16] • When counselling patients, pharmacists should emphasize that the localized action of topical roflumilast allows for effective treatment at the application site, without the same risk of systemic AEs observed with oral roflumilast use [expert opinion]
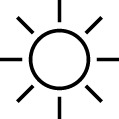 Will topical roflumilast affect my skin sensitivity to the sun or other environmental factors?	• Increased skin sensitivity to the sun or other environmental factors were not observed with the use of topical roflumilast [17, 19, 20]
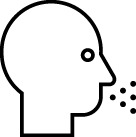 What if my patient experiences an allergic reaction to topical roflumilast?	• Patients experiencing signs of an allergic reaction, such as hives, difficulty breathing, or swelling of the face, lips, tongue, or throat​, should discontinue use and immediately seek medical care
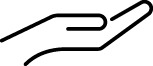 Is thinning of the skin possible with the use of topical roflumilast?	• Thinning of the skin has not been reported with topical roflumilast use in clinical trials [17, 19, 20]
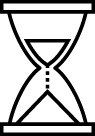 Are there any long-term AEs associated with the use of topical roflumilast?	• No new long-term safety signals associated with topical roflumilast use have been identified in available studies [23, 34]
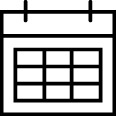 Can the use of topical roflumilast lead to resistance or decreased effectiveness over time?	• There is no known incidence of tachyphylaxis (loss of response) with long-term topical roflumilast use [23]
Drug interactions	
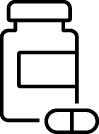 Can topical roflumilast interact with other medications I am currently taking?	• There have been no studies to date examining drug-drug interactions with topical roflumilast [16] • Co-administration of oral roflumilast with systemic CYP3A4 inhibitors (e.g., erythromycin, ketoconazole), CYP1A2 inhibitors (e.g., fluvoxamine), or dual inhibitors (e.g., enoxacin, cimetidine) may increase roflumilast systemic exposure and lead to increased adverse reactions [16] • Based on the bioavailability of topical roflumilast [16], the expectation is that these interactions will be limited with topical use. However, until more guidance is available, patients should be advised of the increased potential for AEs when co-administering topical roflumilast with CYP3A4 inhibitors, CYP1A2 inhibitors, or dual CYP3A4/CYP1A2 inhibitors [expert opinion] • Health professionals should refer to UpToDate® LexidrugTM for updated interaction information and consider potential increases in drug efficacy due to pharmacokinetic interactions [expert opinion]
Contraindications and special populations	
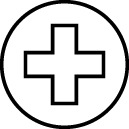 Are there any specific contraindications or conditions that would make topical roflumilast inadvisable?	Pregnant women • While topical roflumilast has no known risk in pregnancy, there are no adequate trials of its use in pregnant women to confirm that it is safe • It is recommended that topical roflumilast should not be used during pregnancy [16] Breastfeeding • There are no data on the presence of topically applied roflumilast in human breast milk, its effects on the breastfed infant, or its effects on milk production [16]. As a result, the safety of topical roflumilast during breastfeeding has not been established. Use during breastfeeding should be approached with caution, and alternative treatments with established safety profiles may be preferred. Clinical judgement should be used to weight the potential benefits of treatment against the unknown risks to the infant [expert opinion] Hepatic impairment • Hepatic impairment significantly increases total PDE4 inhibition following oral roflumilast administration. While there is currently no data to support a similar effect with topical roflumilast, it is recommended that its use still be avoided in patients with moderate-to-severe hepatic impairment (Child-Pugh B or C) to ensure that patients are not exposed to unnecessary risks • It is important to consider the risk of systemic absorption in patients with extensive psoriasis/seborrheic dermatitis and hepatic impairment. Extensive skin involvement may require the application of larger quantities of roflumilast, potentially increasing the risk of systemic absorption, which could be further compounded by hepatic insufficiency [expert opinion] • In cases of mild psoriasis, atopic dermatitis, or seborrheic dermatitis, topical roflumilast may still be considered if no alternative treatments are viable, but only with stringent monitoring • Topical roflumilast is not contraindicated in patients with mild hepatic impairment, though general clinical caution is advised [expert opinion] • If uncertain about the severity of a patient’s hepatic status, consultation with a hepatologist is recommended before prescribing roflumilast
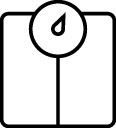 What are the weight loss effects associated with the use of topical roflumilast?	Mild weight loss was noted in 4% of patients treated with roflumilast cream 0.3% compared with 2.3% in the vehicle group [16]. Weight loss was not clinically significant and did not lead any subject to withdraw from the trial. A patient that reports unexplained weight loss of 10% of total baseline body weight should discontinue therapy and seek medical assessment [expert opinion]

Statements are based on topical clinical trial data, product monograph information, extrapolation from oral data and/or expert opinion, as indicated.

### Differences in the pharmacokinetic profiles between topical and oral roflumilast

The difference in safety profiles between topical and oral roflumilast can be attributed to distinct differences in their pharmacokinetic profiles. The pharmacokinetic properties of the oral, cream, and foam formulations have been extensively evaluated, providing evidence to support the more favourable safety and tolerability profile observed with topical roflumilast.

Oral roflumilast exhibits an absolute bioavailability of approximately 79% [35, 36]. It is rapidly absorbed, with a single 500 mcg dose reaching peak plasma concentrations within about one hour of intake [21]. It is extensively metabolized in the liver, primarily by cytochrome P450 isozymes CYP3A4 and CYP1A2, to form its active metabolite, roflumilast *N-oxide,* which reaches a peak plasma concentration within 11 h following a single dose [21]. Although roflumilast *N-oxide* is approximately 3-fold less potent than roflumilast, its total systemic exposure, measured by the area under the plasma concentration-time curve (AUC) is approximately 12-fold greater, accounting for roughly 90% of roflumilast’s overall pharmacological effects [21, 35, 36].

Conversely, roflumilast cream 0.3% exhibits a bioavailability of 1.5% and is slowly absorbed, leading to steady-state plasma concentrations with low peak-to-trough fluctuation [22]. Skin concentrations were shown to be 61.8- and 126-fold higher relative to the plasma following application of 0.5% and 0.15% cream, respectively, while concentrations of roflumilast *N-oxide* were 30-fold lower than circulating plasma concentrations [37]. These data indicate that there is low systemic exposure following topical application and no significant conversion of roflumilast to its *N-oxide* metabolite in the skin, with its pharmacological effects likely due to local PDE4 inhibition by the parent compound [22].

**FIGURE 1:**
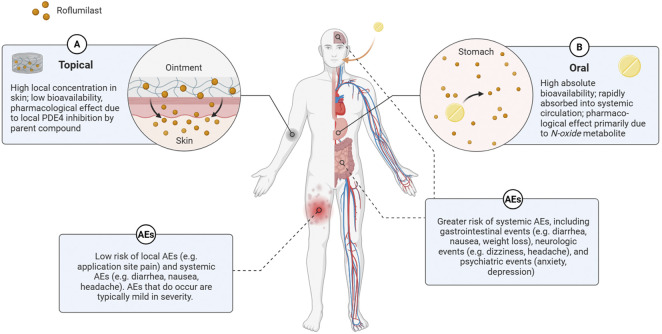
Impact of Route of Administration on the Pharmacokinetic and Adverse Event Profiles of Roflumilast. Topical (A) and oral (B) administration of roflumilast have different pharmacokinetic and adverse event (AE) profiles. Orange circles symbolize roflumilast molecules in both administration pathways. Created with Biorender.com.

The differences in pharmacokinetic profiles between topical and oral formulations underscore the significance of the route of administration on the systemic impact of roflumilast (Figure 1). These pharmacokinetic differences provide important context for interpreting safety findings, particularly where current recommendations are based on oral data rather than direct evidence from topical use.

## Discussion

Pharmacists are confronted with a long list of potential AEs, contraindications, and drug-drug interactions when dispensing topical roflumilast; however, since the majority of these are based on studies of the oral formulation and have not been directly evaluated with topical use, it is vital that the appropriate context is provided when addressing patient concerns about the potential safety risks. To assist with managing these conversations, possible questions that pharmacists may encounter from patients, as well as questions that pharmacists may have themselves, are provided in Table 1, along with practical guidance to address them. The goal is to ensure that patients are well-equipped to make informed decisions about their treatment. By providing clear and accurate information about the relevant safety risks, pharmacists can manage patient expectations and help to alleviate unnecessary anxiety. While the safety risks associated with oral roflumilast should not be dismissed, it is important to recognize that these may be less of a concern with topical use due its targeted action and low systemic exposure observed in clinical pharmacokinetic studies.

Collaboration between pharmacists and dermatologists is essential for optimizing treatment outcomes with topical roflumilast. Establishing clear communication channels allows for timely consultations, accurate information sharing, and shared decision-making, which is especially important in regions where pharmacists have an expanded scope of practice that allows them to initiate treatment. It also helps to ensure that messaging about the potential safety risks of topical roflumilast remains consistent between pharmacists and dermatologists.

While the safety information in the product monograph for topical roflumilast is largely derived from oral studies, key pharmacokinetic differences suggest a more favourable safety profile with topical application. The limited systemic absorption of topical roflumilast is expected to reduce the likelihood of systemic adverse events commonly observed with oral use. However, this has not been directly evaluated in all settings and should be interpreted with caution and clinical judgement (e.g., body surface area involved).

Importantly, several limitations remain. There is currently a lack of long-term safety data, particularly from real-world use, and no formal drug-drug interaction studies have been conducted with topical roflumilast. In addition, evidence in special populations (e.g., hepatic impairment, pregnancy, and broader pediatric populations) is limited. As a result, many safety considerations for topical roflumilast continue to rely on extrapolation from oral data, which may not fully reflect the relative risk profile associated with topical use. Although this review is informed by the Canadian product monograph, the pharmacokinetic principles and clinical trial data discussed are broadly applicable across jurisdictions where topical roflumilast is approved; however, regional differences in regulatory labelling and clinical practice should be considered.

Pharmacists play a crucial role in patient education, ensuring that the safety information is communicated in a way that contextualizes the risks based on the route of administration. Collaboration and alignment of communication between pharmacists and dermatologists is essential to optimizing treatment outcomes and reinforcing accurate safety messaging. Continued pharmacovigilance and real-world data collection will be important to further characterize the long-term safety profile of topical roflumilast, clarify potential interaction risks, and inform its use across broader patient populations.
