# Supplementary material for: Practical considerations for managing patients being treated with topical roflumilast
Source: J Pharm Pharm Sci. 2026 May 8;29:16026. doi: 10.3389/jpps.2026.16026 (PMC13194079; doi:10.3389/jpps.2026.16026)
Supplement: Supplementary file 1 [file Supplementaryfile1.docx]

**Supplementary Table 1.** Examples of Drugs with Both Oral and Topical Formulations.

| Drug | Drug Class | Oral Use | Topical Use |
| --- | --- | --- | --- |
| Ruxolitinib | Janus kinase inhibitor | Splenomegaly and/or associated symptoms in adults with myelofibrosis; control of hematocrit in adults with polycythemia vera (1) | Nonsegmental vitiligo and mild-to-moderate atopic dermatitis (2) |
| Tacrolimus | Calcineurin inhibitor | Prophylaxis of organ rejection in adults receiving allogeneic kidney and liver transplants (3) | Moderate-to-severe atopic dermatitis (4) |
| Diclofenac | Non-steroidal anti-inflammatory drug | Relief of pain and inflammation in various conditions, including rheumatoid arthritis and osteoarthritis (5), migraines (6), dysmenorrhea (7), and musculoskeletal/soft tissue trauma (7) | Localized pain relief associated with osteoarthritis (8) and muscle/joint injuries (9); treatment of inflammation following cataract surgery and in non-penetrating wounds (10); actinic keratosis (11) |
| Dapsone | Sulfone antibiotic | Leprosy, dermatitis herpetiformis, actinomycotic mycetoma (12) | Acne vulgaris (13) |
| Ketoconazole | Imidazole antifungal | Serious or life-threatening systemic fungal infections (14) | Tinea pedis (athlete’s foot), tinea corporis (ringworm), tinea cruris (jock itch), tinea versicolor (pityriasis), cutaneous candidiasis, seborrheic dermatitis, and dandruff (15,16) |
| Minoxidil | Antihypertensive/ vasodilating agent | Severe hypertension (17) | Androgenetic alopecia (18) |
| Clindamycin | Lincosamide antibiotic | Various bacterial infections (19) | Acne vulgaris (20) |

**Supplementary Table 2**. Adverse Reactions Reported in ≥1% of Patients Treated with Topical Roflumilast Cream 0.3% for 8 Weeks, Topical Roflumilast Cream 0.15% for 8 Weeks, Topical Roflumilast Foam 0.3% for 8 Weeks, and 500 mcg Oral Roflumilast for up to 12 Months in Phase 2/3 Clinical Trials.

| Adverse Effect | Roflumilast Cream 0.3% (n=576) (21,22)  *n (%)* | Roflumilast Cream 0.15% (n = 885) (22,23)  *n (%)* | Roflumilast Foam 0.3%  (n= 458) (22,24,25)  *n (%)* | Oral Roflumilast (n=5766) (26–29)  *n (%)* |
| --- | --- | --- | --- | --- |
| Cardiac | | | | |
| Supra-ventricular arrhythmia^a^ | – | – | – | 71 (1.2) |
| Gastrointestinal | | | | |
| Abdominal Pain | – | – | – | 240 (4.2) |
| Diarrhea^b,c^ | 18 (3.1) | 13 (1.5) | – | 668 (11.6) |
| Gastritis | – |  | – | 65 (1.1) |
| Nausea^b,c,d^ | 7 (1.2) | 17 (1.9) | 6 (1.3) | 297 (5.2) |
| Vomiting^c^ | – | 13 (1.5) | – | 76 (1.3) |
| General disorders | | | | |
| Fatigue | – | – | – | 66 (1.1) |
| Infections and infestations | | | | |
| Upper respiratory tract infection^b^ | 6 (1.0) | – | – | – |
| Urinary tract infection^b^ | 6 (1.0) | – | 6 (1.3) | – |
| Nasopharyngitis^d^ | – | – | 7 (1.5) | – |
| COVID-19 | – | – | 12 (2.6) | – |
| Metabolism & nutrition | | | | |
| Decrease appetite | – | – | – | 165 (2.9) |
| Weight decreased^a^ | 23 (4.0) | – | – | 394 (6.8) |
| Musculoskeletal and connective tissue | | | | |
| Back pain | – | – | – | 189 (3.3) |
| Muscle spasms | – | – | – | 112 (1.9) |
| Nervous system | | | | |
| Dizziness^a^ | – | – | – | 177 (3.1) |
| Headache^b,c,d^ | 14 (2.4) | 4 (2.9) | 5 (1.1) | 266 (4.6) |
| Tremor^a^ | – | – | – | 98 (1.7) |
| Psychiatric | | | | |
| Anxiety^a^ | – | – | – | 80 (1.4) |
| Depression^a^ | – | – | – | 73 (1.3) |
| Insomnia^a^ | 8 (1.4) | – | – | 168 (2.9) |
| Vascular | | | | |
| Hypertension | 9 (1.6) | – | – | – |
| Skin and subcutaneous tissue | | | | |
| Application site pain^b,c^ | 6 (1.0) | 13 (1.5) | – | – |
| Contact dermatitis^f^ | – | – | 6 (1.3) | – |
| Discontinuation due to AEs | 6 (1.0) | 14 (1.6) | – | 545 (14.7)^e^ |

^a^ AEs discussed in the product monograph for topical roflumilast based on studies of the oral formulation, though rates are not specifically mentioned.

^b^ AEs included in the product monograph for topical roflumilast as being associated with topical use of 0.3% cream.

**^c^** AEs included in the product monograph for topical roflumilast as being associated with topical use of 0.15% cream.

^d^ AEs included in the product monograph for topical roflumilast as being associated with topical use of 0.3% foam.

^e^ Pooled rates from six pivotal phase 3 clinical trials (n=3701)(27–29)

^f^ All events of contact dermatitis were deemed unrelated to treatment and did not result in an interruption of drug application or dose change.

**References**

1. Novartis Pharmaceuticals Canada Inc. Jakavi® (ruxolitinib phosphate) product monograph. Health Canada Drug Product Database. 2022 [cited 2025 Feb 9]. Available from: pdf.hres.ca/dpd_pm/00065160.PDF

2. Incyte Corporation. Opzelura® (ruxolitinib cream) product monograph. Health Canada Drug Product Database. 2024 [cited 2025 Feb 9]. Available from: pdf.hres.ca/dpd_pm/00077370.PDF

3. Astellas Pharma Canada Inc. Advagraf® (tacrolimus extended-release capsules) product monograph. Health Canada Drug Product Database. 2024 [cited 2025 Feb 9]. Available from: www.astellas.com/ca/system/files/advagraf_pm_hc-approved_eng_23sep2024.pdf

4. LEO Pharma Inc. Protopic® (tacrolimus ointment) product monograph. Health Canada Drug Product Database. 2022 [cited 2025 Feb 9]. Available from: mc-df05ef79-e68e-4c65-8ea2-953494-cdn-endpoint.azureedge.net/-/media/corporatecommunications/canada/therapeutic-expertise/dermatology/our-products/eng/protopicpmenglish27jun2022.pdf?rev=89bd390f97b7429fabab86d8231a2a06&hash=A80BDA5290BDD48CC6BC72E72A1FDA78

5. Pfizer Canada ULC. Arthrotec® 50/75 (diclofenac sodium and misoprostol enteric-coated tablets) product monograph. Health Canada Drug Product Database. 2024 [cited 2025 Feb 9]. Available from: pdf.hres.ca/dpd_pm/00074559.PDF

6. Aralez Pharmaceuticals Canada Inc. Cambia® (diclofenac potassium powder for oral solution) product monograph. Health Canada Drug Product Database. 2021 [cited 2025 Feb 9]. Available from: pdf.hres.ca/dpd_pm/00063848.PDF

7. Sandoz Canada Inc. Sandox Diclofenac Rapide (diclofenac potassium) product monograph. Health Canada Drug Product Database. [cited 2025 Feb 9]. Available from: pdf.hres.ca/dpd_pm/00065682.PDF

8. PHARMASCIENCE INC. Diclofenac (diclofenac sodium solution) product monograph. Health Canada Drug Product Database. 2022 [cited 2025 Feb 9]. Available from: pdf.hres.ca/dpd_pm/00066637.PDF

9. GlaxoSmithKline Consumer Healthcare ULC. Voltaren Emulgel (diclofenac diethylamine gel) product monograph. Health Canada Drug Product Database. 2023 [cited 2025 Feb 9]. Available from: pdf.hres.ca/dpd_pm/00071716.PDF

10. Sandoz Canada Inc. Sandoz Diclofenac Ophtha (diclofenac sodium ophthalmic solution) product monograph. Health Canada Drug Product Database. 2023 [cited 2025 Feb 9]. Available from: pdf.hres.ca/dpd_pm/00071592.PDF

11. Alembic Pharmaceuticals Inc. Diclofenac sodium (diclofenac sodium topical gel) prescribing information. Health Canada Drug Product Database. 2025 [cited 2025 Feb 9]. Available from: dailymed.nlm.nih.gov/dailymed/drugInfo.cfm?setid=30c3c3c5-b4f3-4321-ae25-b2c588ad0551#section-1

12. Marcan Pharmaceuticals Inc. Mar-Dapsone (dapsone tablets) prescribing information. Health Canada Drug Product Database. 2018 [cited 2025 Feb 10]. Available from: pdf.hres.ca/dpd_pm/00047474.PDF

13. Bausch Health Canada Inc. Aczone® (dapsone gel) product monograph. Health Canada Drug Product Database. 2020 [cited 2025 Feb 10]. Available from: pdf.hres.ca/dpd_pm/00057223.PDF

14. APOTEX INC. Ketoconazole (ketoconazole tablets) product monograph. Health Canada Drug Product Database. 2021 [cited 2025 Feb 10]. Available from: pdf.hres.ca/dpd_pm/00060666.PDF

15. TaroPharma. Ketoderm (ketoconazole cream) product monograph. Health Canada Drug Product Database. 2003 [cited 2025 Feb 10]. Available from: pdf.hres.ca/dpd_pm/00000085.PDF

16. Kramer Laboratories Inc. Nizoral® (ketoconazole shampoo) product monograph. Health Canada Drug Product Database. 2019 [cited 2025 Feb 10]. Available from: pdf.hres.ca/dpd_pm/00050002.PDF

17. TM Pharmacia & Upjohn Company LLC. Loniten® (minoxidil tablets) product monograph. Health Canada Drug Product Database. 2013 [cited 2025 Feb 10]. Available from: pdf.hres.ca/dpd_pm/00022732.PDF

18. Johnson & Johnson Inc. Rogaine® (minoxidil foam) product monograph including patient medication information. Health Canada Drug Product Database. [cited 2025 Feb 10]. Available from: pdf.hres.ca/dpd_pm/00064467.PDF

19. Pfizer Canada ULC. Dalacin® C Flavoured Granules (clindamycin palmitate hydrochloride) product monograph including patient medication information. Health Canada Drug Product Database. 2022 [cited 2025 Feb 10]. Available from: pdf.hres.ca/dpd_pm/00064286.PDF

20. Bausch Health Canada Inc. Clinda-T® (clindamycin phosphate topical solution) product monograph. Health Canada Drug Product Database. 2022 [cited 2025 Feb 10]. Available from: pdf.hres.ca/dpd_pm/00065052.PDF

21. Lebwohl MG, Kircik LH, Moore AY, Stein Gold L, Draelos ZD, Gooderham MJ, et al. Effect of Roflumilast Cream vs Vehicle Cream on Chronic Plaque Psoriasis: The DERMIS-1 and DERMIS-2 Randomized Clinical Trials. JAMA. 2022;328(11):1073–84.

22. Arcutis Canada Inc. Arcutis Canada, Inc. 2025 [cited 2025 Mar 19]. p. 1–36 Product Monograph Including Patient Medication Information: ZORYVE® (roflumilast). Available from: https://pdf.hres.ca/dpd_pm/00078853.pdf

23. Simpson EL, Eichenfield LF, Alonso-Llamazares J, Draelos ZD, Ferris LK, Forman SB, et al. Roflumilast Cream, 0.15%, for Atopic Dermatitis in Adults and Children. JAMA Dermatol. 2024 Nov 1;160(11):1161.

24. Blauvelt A, Draelos ZD, Stein Gold L, Alonso-Llamazares J, Bhatia N, DuBois J, et al. Roflumilast foam 0.3% for adolescent and adult patients with seborrheic dermatitis: A randomized, double-blinded, vehicle-controlled, phase 3 trial. J Am Acad Dermatol. 2024;90(5):986–93.

25. Zirwas MJ, Draelos ZD, Dubois J, Kircik LH, Moore AY, Stein Gold L, et al. Efficacy of Roflumilast Foam, 0.3%, in Patients With Seborrheic Dermatitis: A Double-blind, Vehicle-Controlled Phase 2a Randomized Clinical Trial. JAMA Dermatol [Internet]. 2023 [cited 2025 Feb 4];159(6):613–20. Available from: https://pubmed.ncbi.nlm.nih.gov/37133856/

26. AstraZeneca Canada Inc. Mississauga, ON: AstraZeneca Canada Inc. 2021 [cited 2025 Feb 10]. DAXAS® (roflumilast tablets) product monograph. Available from: https://www.astrazeneca.ca/content/dam/az-ca/downloads/productinformation/daxas-product-monograph-en.pdf

27. Fabbri LM, Calverley PM, Izquierdo-Alonso JL, Bundschuh DS, Brose M, Martinez FJ, et al. Roflumilast in moderate-to-severe chronic obstructive pulmonary disease treated with longacting bronchodilators: two randomised clinical trials. The Lancet [Internet]. 2009 [cited 2025 Feb 10];374(9691):695–703. Available from: https://pubmed.ncbi.nlm.nih.gov/19716961/

28. Rennard SI, Calverley PMA, Goehring UM, Bredenbröker D, Martinez FJ. Reduction of exacerbations by the PDE4 inhibitor roflumilast--the importance of defining different subsets of patients with COPD. Respir Res [Internet]. 2011 [cited 2025 Feb 10];12(1). Available from: https://pubmed.ncbi.nlm.nih.gov/21272339/

29. Calverley PM, Rabe KF, Goehring UM, Kristiansen S, Fabbri LM, Martinez FJ. Roflumilast in symptomatic chronic obstructive pulmonary disease: two randomised clinical trials. The Lancet [Internet]. 2009 [cited 2025 Feb 10];374(9691):685–94. Available from: http://www.thelancet.com/article/S0140673609612551/fulltext
